# What We Talk about When We Talk about Unconscious Processing – A Plea for Best Practices

**DOI:** 10.3389/fpsyg.2017.00835

**Published:** 2017-05-23

**Authors:** Marcus Rothkirch, Guido Hesselmann

**Affiliations:** Visual Perception Laboratory, Department of Psychiatry and Psychotherapy, Campus Charité Mitte, Charité – Universitätsmedizin BerlinBerlin, Germany

**Keywords:** consciousness, awareness, unconscious processing, best practices, diversity

## Abstract

In this perspective article, we first outline the large diversity of methods, measures, statistical analyses, and concepts in the field of the experimental study of unconscious processing. We then suggest that this diversity implies that comparisons between different studies on unconscious processing are fairly limited, especially when stimulus awareness has been assessed in different ways. Furthermore, we argue that flexible choices of methods and measures will inevitably lead to an overestimation of unconscious processes. In the concluding paragraph, we briefly present solutions and strategies for future research. We make a plea for the introduction of “best practices,” similar to previous attempts to constitute practicing standards for functional magnetic resonance imaging (fMRI) and electroencephalography (EEG).

## Introduction

To what extent are our actions and thoughts determined by “unconscious” influences, that is, by influences that we ourselves are not aware of? Sixty years after the infamous marketing campaign launched by James Vicary in 1957, who claimed that unconsciously flashed messages can affect observers’ consumption behavior (Karremans et al., 2006), this question has lost nothing of its fascination. What is more, the development of scientific methods, like neuroscientific instruments or psychophysical “blinding” techniques, has made it possible to assess unconscious processes from different perspectives and within a wide range of contexts. There is, however, a downside to this multifaceted approach to unconscious processing: It comes along with substantial diversity between scientific studies.

In the following sections, we will first provide a brief overview of aspects in consciousness research that are affected by this diversity. Afterward, we will outline the risks associated with such divergent approaches. We will conclude with a section on practical recommendations for future research.

## Diversities Regarding Different Experimental Aspects

### Diversity in Suppression Techniques

Today, a wide range of psychophysical paradigms exist to experimentally manipulate sensory awareness, particularly within the visual domain (Bachmann et al., 2007). Such psychophysical “blinding” methods can evoke a transient blindness in neurologically intact observers, i.e., they can be used to render a physically presented target stimulus (or, features of it) invisible, in most cases for a limited amount of time. It is important to keep in mind that the available paradigms vary with respect to what types of stimuli can be suppressed from awareness, and how effective the suppression is, for instance in terms of duration and predictability of suppression onset and offset (Kim and Blake, 2005). But there is yet another level of diversity related to the “blinding” methods that has only recently begun to be studied systematically. In 2014, a Frontiers Research Topic [“Invisible, but how?”; edited by (Dubois and Faivre, 2014)] addressed the issue of the inherent differences in the amount of information let through by different suppression techniques. Put simply, it is not enough to know that a stimulus has been invisible, but it is crucial to also know how exactly invisibility was achieved, because it has become apparent that different levels of suppression depth are associated with different suppression methods. Recently, this notion has been formalized into a functional hierarchy of unconscious visual processing (Breitmeyer, 2014). Eventually, such a functional hierarchy will allow to formulate predictions about the level of unconscious processing that can be expected in a specific experimental setup (Breitmeyer, 2015).

In the absence of such prior assumptions on the depth of visual suppression associated with a specific paradigm, every new study reporting high-level unconscious processing is equally weighted, independently of the applied suppression method. When accumulating evidence across single studies, this might lead to the wrong conclusion that unconscious perception is unlimited. However, it is known that different suppression methods are associated with different levels of suppression. Therefore, for example, the evidence presented for unconscious processing under CFS should be stronger, as a plausible prior assumption could be that CFS leads to a relatively high level of suppression. Ideally, the functional hierarchy would be based on cumulative research strategies, and incorporate not only studies that directly compared results from different methods with each other (Izatt et al., 2014; Peremen and Lamy, 2014a), but also take data from neuroimaging studies into account (Fogelson et al., 2014; Ludwig et al., 2016).

### Diversity in Awareness Measures

The question of how to optimally measure awareness in experiments on unconscious processing has been a long-standing one, and the issue is still much debated (e.g., Merikle and Reingold, 1990; Kunimoto et al., 2001; Schmidt and Vorberg, 2006; Sandberg et al., 2010). While criteria for valid awareness measures have been formulated (Shanks and John, 1994; De Houwer et al., 2009; Newell and Shanks, 2014), there is no accepted “gold standard.” We will briefly outline the diversity of frequently applied measures of awareness, but this overview is not meant to be exhaustive. One crucial differentiation is the distinction between objective and subjective measures of awareness. According to the objective awareness criterion, forced-choice detection or discrimination performance above chance level indicates stimulus awareness, while performance at chance level indicates its absence. Subjective measures of awareness, on the other hand, are based on participants’ metacognitive judgements on their own mental states (Lau and Rosenthal, 2011). Usually participants perform such judgments either on their own experience of the stimulus, or on their accuracy in a discrimination task. In the first case, participants are required to rate the visibility of the stimulus, either on a larger (Sergent and Dehaene, 2004) or smaller scale (Ramsøy and Overgaard, 2004). In the second case, participants have to evaluate how confident they felt with their response in a previously performed discrimination task (e.g., Rothkirch et al., 2012). The pros and cons of objective and subjective awareness measures have been summarized elsewhere (e.g., Hesselmann, 2013). A further distinction can be made between filtering measures and aggregate measures of awareness. If behavioral reports are provided on a trial-by-trial basis during the main experiment (“online”), filtering allows the experimenter to post-select subsets of trials for further analysis (e.g., “seen” vs. “not seen”). By contrast, aggregate measures allow inferences only about blocks of trials and thus depend on more than a single trial (e.g., percent correct, d’). Behavioral reports may be recorded either after a block of trials or in a separate control experiment (“offline,” or “two-task design”). If a pre-defined criterion, such as performance at chance level, is satisfied, the experimenter infers that stimulus awareness was absent during the main experiment (or, in a given block of trials). Importantly, diversity does not end here, and the devil is in the details. For example, it has been shown that the exact composition of a block of trials (e.g., blocks with weakly and fully visible stimuli, vs. blocks with only weakly visible stimuli) can influence the experienced level of awareness [(Lin and Murray, 2014); also see (Pratte and Rouder, 2009)]. Levels of stimulus awareness may also change in longer experiments, e.g., awareness can increase across trials due to perceptual learning (Schwiedrzik et al., 2011; Ludwig et al., 2013). Finally, the presence or absence of trial-by-trial awareness reports can in turn influence the effect of interest, e.g., response priming (Peremen and Lamy, 2014b).

### Diversity in Statistical Analysis

We will briefly illustrate the diversity in the statistical analysis of awareness test data by an example. It is not uncommon that participants perform a two-alternative forced-choice (2AFC) discrimination task in a control experiment, either in addition to a subjective awareness measure (e.g., Hesselmann et al., 2016), or without any further awareness tests during the main experiment (e.g., Koechlin et al., 1999; Mattler and Palmer, 2012). To show that participants did not perceive the presented stimuli, researchers then often apply null hypothesis significance testing (NHST) on the 2AFC data, either at the subject level or at the group level. Alternatively, the researcher may choose to apply a cutoff, e.g., consider 2AFC performance below 60% as indicative of an absence of stimulus awareness. It is well understood that when the NHST procedure is underpowered (or, the power is unknown), the experimenter risks to falsely accept the null hypothesis, i.e., conclude that there is no deviation from chance level when there actually is a deviation (Vadillo et al., 2016b). For example, the number of trials included in the awareness test may determine whether participants are categorized as aware or unaware, simply because tests comprising more trials also yield more sensitive measures. There have been suggestions to avoid the problem of type II errors by using equivalence tests and equivalence confidence intervals (Overgaard et al., 2013). A challenge related to such equivalence tests, however, is the *a priori* definition of a boundary around chance level that is still considered acceptable to indicate unawareness (Lin and Murray, 2015). Recently, Bayesian statistics have more frequently been applied to establish chance performance (e.g., Dienes, 2015; Sand and Nilsson, 2016). The details of this approach are beyond the scope of this perspective article, but it is important to keep in mind that Bayesian statistics are inherently diverse too. For example, when using Bayes factors to estimate the evidence in favor of the null model, there are different options for specifying the predictions of the alternative model (i.e., the case where participants saw the stimulus). One particularly relevant feature of the Bayesian approach seems to be sequential sampling, i.e., the strategy to collect more data until the evidence in favor of one model is considered as conclusive. Beyond the question of NHST or Bayesian statistics, *post hoc* selection of data has frequently been used, for example when residual stimulus visibility greatly varies between participants in experiments using interocular suppression (e.g., Sklar et al., 2012). The idea is to perform the main statistical analysis on a *post hoc* selection of the recorded data, e.g., by excluding participants whose subjective or objective behavioral reports indicated stimulus awareness, or by exclusively analyzing “not seen” trials. It has, however, been criticized to interpret “not seen” trials purely in isolation (Schmidt, 2015). Instead, these trials should be contrasted against seen trials, i.e., trials in which the suppression of the critical stimulus was not successful (cf. Madipakkam et al., 2015). Furthermore, especially in cases in which a large number of trials or participants are excluded, data analysis can resemble some form of extreme group analysis. Due to regression to the mean such an analysis strategy can yield a statistical bias in the selected sample and let the researcher erroneously assume unawareness in the selected participants (Shanks, 2016). The magnitude of regression to the mean in a data set is indicated by the correlation between the awareness measure and the measured effect. In this context, a model taking only into account regression to the mean can function as a null model against which the experimental effect can be tested. It has to be noted, however, that the extent to which the experimental effect differs from such a null model neither conclusively demonstrates nor precludes an unconscious effect.

### Diversity in Experimental Setting

In a typical psychological experiment, stimuli are presented to participants who are asked to elicit a particular response to these stimuli. If such an experiment is intended to investigate unconscious processes, the unconscious dimension could be related to different aspects of the experimental setup. Firstly, participants could be unconscious of the presented stimulus, which is usually achieved by some form of masking (Kim and Blake, 2005). The second category is constituted by studies in which the putatively unconscious process pertains to the relationship between the stimulus and participants’ behavior. This is, for instance, typically the case in studies on social priming (Bargh, 2016). Finally, the behavioral response or sequence of responses elicited by the participants might be unconscious, as in studies focusing on learning processes (Destrebecqz and Cleeremans, 2001).

While in cognitive psychology the focus lies primarily on the unawareness of the stimuli, in social psychology the relation between stimuli and observers’ behavior is often the primary target (Doyen et al., 2014). Especially in the latter case, however, it proves difficult to determine whether the process of interest is indeed unconscious. As pointed out by (Stafford, 2014), for instance, the degree to which observers lack knowledge about associations between stimuli and their behavior that is required to be considered ‘unconscious’ is conceptually elusive. An ostensible remedy often chosen in the face of this predicament is then to mask the stimulus such that it cannot be consciously perceived anymore. This demonstrates that the different approaches to study unconscious processes are often used as if they were interchangeable, although they might target distinct processes.

Remarkably, it is widely accepted, on the one hand, that consciousness is not a unitary process, but that there are instead distinct modes of ‘consciousness,’ which, for instance, implies that observers might have conscious knowledge about one aspect of a particular stimulus while lacking conscious access to other aspects of that same stimulus (Zeki and Bartels, 1998; Navajas et al., 2014). In contrast, however, unconsciousness is sometimes treated as a singular process that does not require a further differentiation, although the term “unconscious” is not unitary either (Moors and De Houwer, 2006).

## Consequences of the Outlined Diversities

As summarized above, researchers have several options to study and identify unconscious processes, ranging from the experimental design to the statistical analysis of the acquired data. While this patchwork of approaches could be deemed beneficial in the sense that it entails a more extensive overview of the phenomenon of unconscious processing, we argue that it more likely has the opposite effect. It should be noted that the diversities detailed above have distinct implications. On the one hand, they can have direct consequences on the interpretability of findings from single studies, which can, in severe cases, imply that participants were actually not unaware of the presumed unconscious process. Such consequences are especially related to the diversities discussed in section “Diversity in Awareness Measures and Diversity in Statistical Analysis.” On the other hand, the diversities regarding the experimental setting (see section Diversity in Experimental Setting) and the suppression techniques (see section Diversity in Suppression Techniques) have less impact on single studies, but play a more important role at the conceptual level, that is, when the findings across several studies are intended to be integrated into a general framework or model of (un)consciousness.

Specifically, studies differ with respect to the way awareness or unawareness, respectively, is assessed and statistically analyzed. This means that different criteria are set to determine whether participants are aware or unaware of a particular stimulus or process. For a given study, it is thus not sufficient to simply know *whether* participants were unaware, but instead according to *which criterion* participants were unaware, since it cannot be precluded that other criteria might indicate participants’ awareness of the critical stimulus or process. This, however, limits the comparability between different studies, especially when awareness has been assessed in different ways. For example, two functional magnetic resonance imaging (fMRI) studies have investigated neural activity in visual areas when images of objects were rendered invisible with CFS (Fang and He, 2005; Hesselmann and Malach, 2011). In one study (Fang and He, 2005), participants were asked to report whether they perceived any shape or object in the preceding block of trials. In the other case (Hesselmann and Malach, 2011), participants provided trial-by-trial visibility ratings on a 3-point scale. It could be argued that only a limited comparison of the effect of interest is possible between the two studies, since evidence for participants’ unawareness was based on different types of responses.

Another critical aspect that especially pertains to the assessment of observers’ awareness is the flexibility in data analysis based on the vast amount of awareness definitions. Simply put, if researchers are interested in demonstrating that observers were unaware they could choose to report a particular analysis that indeed speaks in favor of observers’ unawareness, while neglecting that other criteria might have indicated awareness. For example, when faced with the divergence of subjective and objectives measures in some participants, researchers may argue in terms of “blindsight” in normal observers (Hesselmann et al., 2011), criticize the underlying assumption of different objective and subjective thresholds (Peters and Lau, 2015), or choose one or the other measure to demonstrate observers’ unawareness. Such a flexible choice could overstretch the concept of unawareness and consequently lead to an overestimation of unconscious processes, as the researcher has the “freedom” to report only the particular analysis that confirms their expectation (cf. Simmons et al., 2011).

On a more conceptual level, the vagueness of the terms “awareness” and “consciousness” allows one to operate with them in different contexts (Moors and De Houwer, 2006). If two different studies make reference to “unconscious processes,” the reader may likely assume that similar processes have been studied, although one study may, say, use visually masked stimuli while the other one may focus on “unconscious processes” in response to visible stimuli. Such an inflationary reference to “awareness” and “consciousness” bears the risk of counteracting a differentiation between qualitatively distinct phenomena and may eventually – perhaps even inevitably – lead to the claim that unconscious processes can carry out every fundamental high-level cognitive function that conscious processes can perform [(Hassin, 2013); for a reply (Hesselmann and Moors, 2015)], and thus impede deeper insights into the nature of unconscious processes.

Finally, one can speculate that the outlined diversities also contribute to the current crisis of confidence in psychological research. Some of the findings on unconscious processes, including, among others, effects of social priming and unconscious perception, turned out to be difficult to replicate (Doyen et al., 2012; Hesselmann and Knops, 2014; Moors et al., 2016), show clear signs of bias (Shanks et al., 2015; Vadillo et al., 2016a), or are open to alternative explanations (Stein et al., 2016; Street and Vadillo, 2016).

## Solutions and Strategies for Future Research

One obvious strategy to handle the described diversity is the formulation of explicit guidelines for the assessment and statistical analysis of awareness. Such guidelines may not only help to unify the fragmented approaches that exist to study unconscious processing, but also may serve as a guide for researchers who are not yet acquainted with the peculiarities and pitfalls related to the assessment of observers’ level of awareness. They should, however, not be understood as a discrete set of binding rules. Instead, we propose that the whole field should strive for the establishment of ‘best practices,’ similar to previous attempts to constitute practicing standards for fMRI (Poldrack et al., 2017) and EEG research (Picton et al., 2000). For example, the “21-word-solution” (Simmons et al., 2012) that is intended to make the *post hoc* exclusion of conditions and/or participants transparent, could easily be adopted by the field, given the fact that *post hoc* exclusions may indeed cause serious problems in studies on unconscious processing (Shanks, 2016). Ideally, full transparency of methods should be the norm. Notably, the current diversity between studies will not be completely abolished by such an intervention, but only reduced. The definition of an overarching ‘gold standard’ would not be feasible, since, for instance, the possibilities to assess awareness are often limited by the particular experimental design.

Moreover, it should be acknowledged that qualitatively distinct phenomena are subsumed under the umbrella terms “consciousness” and “unconsciousness.” To increase the understanding of the scope and limits of unconscious processes, however, such a deficient differentiation seems counterproductive. Thus, to reflect and accentuate that research on unconscious processing stems from a variety of experimental settings, a more fine-grained taxonomy of unconscious phenomena seems expedient. For instance, studies in which the processing of visually masked stimuli is investigated could consistently label “unconscious processing” as “subliminal processing” (Dehaene et al., 2006). In contrast, if *supraliminal* information is processed unbeknownst to the observer in an incidental manner, as in the case of “unconscious learning processes,” one could label this as “implicit processing.” The choice of such terms should also be tailored to their underlying assumptions, as in the case of “subliminal” for instance, which could suggest a high-threshold model. Thus, other labels may be more suitable. Especially to keep up with the development of new techniques, methods, and experimental designs, however, such a differentiation is generally advisable.

Finally, we believe that a great service to this field will be done by an adherence to openly available data, so that the results can be reproduced, and the conclusions tested against alternative statistical analyses.

## Author Contributions

MR and GH: Conception of the manuscript, Preparation of a first draft, Contribution of critical input and finalization of the manuscript.

## Conflict of Interest Statement

The authors declare that the research was conducted in the absence of any commercial or financial relationships that could be construed as a potential conflict of interest.
